# Extracellular vesicles from *Listeria monocytogenes*-infected dendritic cells alert the innate immune response

**DOI:** 10.3389/fimmu.2022.946358

**Published:** 2022-09-05

**Authors:** Raúl Izquierdo-Serrano, Irene Fernández-Delgado, Olga Moreno-Gonzalo, Enrique Martín-Gayo, Diego Calzada-Fraile, Marta Ramírez-Huesca, Inmaculada Jorge, Emilio Camafeita, Joaquín Abián, Miguel Vicente-Manzanares, Esteban Veiga, Jesús Vázquez, Francisco Sánchez-Madrid

**Affiliations:** ^1^ Vascular Pathophysiology Area, Centro Nacional Investigaciones Cardiovasculares (CNIC) Carlos III, Madrid, Spain; ^2^ Department of Immunology, Instituto Investigación Sanitaria Hospital Universitario La Princesa (IIS-HUP), Universidad Autónoma de Madrid (UAM), Madrid, Spain; ^3^ Centro de Investigación Biomédica en Red de Enfermedades Cardiovasculares (CIBERCV), Madrid, Spain; ^4^ Biological and Environmental Proteomics, Institut d’Investigacions Biomèdiques de Barcelona, Consejo Superior de Investigaciones Científicas (IIBB-CSIC), Institut d’Investigacions Biomèdiques August Pi i Sunyer (IDIBAPS), Barcelona, Spain; ^5^ Molecular Mechanisms Program, Centro de Investigación del Cáncer and Instituto de Biología Molecular y Celular del Cáncer, Consejo Superior de Investigaciones Científicas (CSIC)-Universidad de Salamanca, Salamanca, Spain; ^6^ Department of Molecular & Cellular Biology, Centro Nacional de Biotecnología, Consejo Superior de Investigaciones Científicas (CNB-CSIC), Madrid, Spain

**Keywords:** *Listeria monocytogenes* infection, dendritic cells (DCs), extracellular vesicles (EVs), immune alert, HDAC6

## Abstract

Communication through cell-cell contacts and extracellular vesicles (EVs) enables immune cells to coordinate their responses against diverse types of pathogens. The function exerted by EVs in this context depends on the proteins and nucleic acids loaded into EVs, which elicit specific responses involved in the resolution of infection. Several mechanisms control protein and nucleic acid loading into EVs; in this regard, acetylation has been described as a mechanism of cellular retention during protein sorting to exosomes. HDAC6 is a deacetylase involved in the control of cytoskeleton trafficking, organelle polarity and cell migration, defense against *Listeria monocytogenes (Lm)* infection and other immune related functions. Here, we show that the protein content of dendritic cells (DCs) and their secreted EVs (DEVs) vary during *Lm* infection, is enriched in proteins related to antiviral functions compared to non-infected cells and depends on HDAC6 expression. Analyses of the post-translational modifications revealed an alteration of the acetylation and ubiquitination profiles upon *Lm* infection both in DC lysates and DEVs. Functionally, EVs derived from infected DCs upregulate anti-pathogenic genes (e.g. inflammatory cytokines) in recipient immature DCs, which translated into protection from subsequent infection with vaccinia virus. Interestingly, absence of Listeriolysin O in *Lm* prevents DEVs from inducing this anti-viral state. In summary, these data underscore a new mechanism of communication between bacteria-infected DC during infection as they alert neighboring, uninfected DCs to promote antiviral responses.

## Introduction

*Listeria monocytogenes* (*Lm*), a facultative-anaerobic, Gram-positive intracellular bacterium, is a foodborne pathogen that can be present in water and raw food or transmitted through contaminated surfaces. *Lm* infection leads in some cases to listeriosis, an infection of the central nervous system (CNS) and the fetal-placental unit, causing serious complications and even abortion (1). During infection, *Lm* enters phagocytic and non-phagocytic eukaryotic cells (2). Once inside the cell, *Lm* escapes the vacuole by avoiding its fusion with the lysosome due to an hemolysin protein, known as Listeriolysin O (LLO) (3). Having left the vacuole, it can induce the formation of actin comets (4) that enable cell-to-cell spread through a process called paracytophagy (5). This mechanism enables *Lm* to escape immune detection. Still, bacterial products activate monocytes in a toll-like receptor (TLR)-dependent manner, inducing differentiation into tumor-necrosis factor (TNF)/-inducible nitric oxide synthase (iNOS)-producing dendritic cells (Tip-DCs) (6). Multiple signaling pathways regulate this process. We recently reported that the association of the cytoplasmic deacetylase HDAC6 (7) with the TLR-adaptor protein MyD88 controls TLR2 signaling to drive innate immune cell activation and bacterial clearance (8). *Hdac6^-/-^* murine bone marrow-derived dendritic cells (BMDCs) displayed defective innate immune responses against intracellular bacteria, particularly *Lm.* Upon *Lm* infection, HDAC6-deficient BMDCs expressed lower levels of interferon (IFN)-related genes and pro-inflammatory cytokines *Lm* (8), which has an impact on innate as well adaptive immune responses.

Bacterial infection elicits large-scale changes in the immune secretome. Some of its components are Extracellular Vesicles (EVs). EVs modulate diverse immune processes including antigen presentation, immune activation, induction of tolerance, or suppression of immune responses (9–12). EVs are classified based on the nature of the cellular compartment they originated and include: exosomes, microvesicles and apoptotic bodies (13). EVs contain proteins, nucleic acids and lipids. EVs membranes possess a characteristic lipid profile with enriched sphingolipids and ceramide (14). In addition, a specific nucleotide motif (GGAG) was found to control the loading of miRNAs into exosomes (15). The sorting and packaging of these components into EVs is highly regulated (15), where post-translational modifications (PTMs) are playing a leading role (16). These molecules play important roles in immune cell-to-cell communication (17). DCs produce EVs (termed DEVs). DEVs present antigens to T cells and may contribute to tumor immunosurveillance and infections (18). For instance, during Dengue Virus Infection immune related molecules sorted to DEVs play an important role for viral neutralization (19).

In this study, we aimed to determine the role of EVs derived from *Lm*-infected DCs in the innate response, and the potential role of HDAC6 in DEV loading under these conditions. Our results show that *Lm* infection induced an elevated production of DEVs. *Lm* infection also altered protein sorting. Interestingly, HDAC6 deletion altered DEV protein sorting, although it did not affect acetylation of DEV-loaded proteins. However, protein ubiquitination was severely altered. Immature DCs treated with DEVs from infected DCs upregulated antiviral and maturation DC genes and promoted an increase in interleukin (IL)-1β secretion. Functionally, the overall expression of antiviral genes led us to choose a virus as a proof of concept. Additionally, we have previously used this strategy to successfully study the effect of EVs cargo proving that T cells prime DCs through the transfer of exosomal DNA, making them more resistant to subsequent viral infections (20). These infection studies lead to the protection of DC from a subsequent viral infection with vaccinia virus (VACV) when pretreated with DEVs derived from *Lm* infection. These data indicate that DEVs are part of a mechanism of cell-to-cell alert between infected DCs and immature DCs mediated by DEVs.

## Materials and methods

### Mice

Mice were housed under specific pathogen-free conditions at the Centro Nacional de Investigaciones Cardiovasculares Carlos III (CNIC) and experiments were approved by the CNIC Ethical Committee for Animal Welfare and by the Spanish Ministry of Agriculture, Food, and the Environment. *Hdac6^-/-^* mice were generated by disruption of the first catalytic domain of HDAC6 (21) and were backcrossed on a C57BL/6 background.

### Antibodies and other reagents

Antibodies and reagents used are listed in **Table S1**.

### Generation of bone marrow-derived dendritic cells (BMDCs)

Mouse BMDCs were obtained through the differentiation of hematopoietic stem cells from bone marrow after culture on non-treated 150mm Petri dishes with RPMI (Sigma-Aldrich) containing 1% Sodium pyruvate (Lonza), Hepes 20mM pH 7.5 (Cambrex), 50μM 2-mercaptoethanol and 10% fetal bovine serum (FBS) (Invitrogen) and supplemented with 20ng/mL recombinant mouse granulocyte-macrophage colony-stimulating factor (GM-CSF) (PeproTech) and 50U/mL penicillin (Lonza), 50μg/mL streptomycin (Lonza) for differentiation. BMDCs were collected at day 9 and were characterized by CD11c^+^MHCII (I-A/I-E)^+^GR-1^-^ by flow cytometry analysis.

### Generation of blast T cells from spleen

Spleen cell suspensions were obtained from mice by grinding organs through a 70 μm cell strainer (Fisher Scientific, 10788201). ACK lysis buffer (Lonza, 10-548E) was used to lysate erythrocytes for 5 min at RT. Splenocytes were cultured on treated flasks at a concentration of 1.5 · 10^6^ cells/mL in complete RPMI-1640 supplemented with 2 μg/mL concanavalin A (Sigma-Aldrich) for TCR signal activation. After 1.5 days cells were washed several times with PBS and cultured at the same concentration in complete RPMI-1640 supplemented with 200 U/mL recombinant IL-2 (STEMCELL, 78036.2) to maintain cell proliferation. Fresh media was added every two days. Blasted splenocytes were collected at day 6 for experiments.

### Bacteria strains

We used the bacteria strains *Listeria monocytogenes* wild type EGD (BUG 600) and its isogenic Listeriolysin O (LLO) deficient mutant Δ*hly* (BUG 3650), provided by Dr. Pascale Cossart (Pasteur Institute, Paris, France). *Lm* was grown overnight in a Brain Heart Infusion Broth (BHI) inverted plate.

### *In vitro Lm*-infection of BMDCs

For infection of BMDCs, a colony of *Lm* was grown in BHI at 37°C with shaking (250rpm) overnight. To assess colony-forming units (CFUs) bacterial growth was meassured by spectrophotometry and used in log-phase (optical density 0.8-1.2 at 600 nm). DCs were incubated or not (infected or non-infected DCs) with *Lm* at a multiplicity of infection (MOI) of 10 for 30 min at 37°C. To determine the effect of intracellular *Lm* alone and avoid future re-infections, extracellular bacteria were killed by treatment with 100 μg/mL gentamicin (Sigma-Aldrich). After 30 min of this treatment, the 0 h post-infection (hpi) is settled down (8, 22). EVs were isolated from cell media at 16 hpi.

### Extracellular vesicles purification

DCs were cultured in RPMI medium supplemented with 10% exosome-depleted FBS (Invitrogen); FBS was depleted of bovine EVs by centrifugation at 100,000 × g for at least 16–20 h. Subsequently, EVs were obtained by serial centrifugation. First, cells were pelleted at 500 × g for 5 min; the resulting supernatant was centrifuged at 2000 × g for 15 min to eliminate cellular debris and dead cells; the collecting supernatant was then ultracentrifugated at 10,000 × g for 30min at 4°C (Beckman Coulter Optima L-100 XP, Beckman Coulter) to remove remaining debris. EVs were pelleted at 100,000 × g for 70 min at 4°C. This pellet was then washed with PBS to remove any remaining cell medium contaminants and ultracentrifugated again, 100,000 × g for 70 min at 4°C. Then, the pellet was suspended in PBS. EVs from 5 to 10 different mice donors were pooled together for each condition. Then, EVs were administered according to the number of producer cells (25 · 10^6^ cells/donor).

### Nanoparticle tracking analysis (NTA)

EV numbers and size distribution were determined by the rate of Brownian motion in a NanoSight LM10 system, which is equipped with fast video capture and particle-tracking software (NanoSight, Amesbury, UK). Samples were diluted before analysis and 0.5 mL of diluted EV fraction was loaded into the chamber of this LM10 unit, and the relative concentration was calculated according to the dilution factor. Data were analyzed with NTA 2.1 software (Nanosight). Samples were analyzed using manual shutter and gain adjustments, which resulted in shutter speeds of 15 or 30 ms, with camera gains between 280 and 560. The detection threshold was kept above 4; blur: auto; minimum expected particle size: 50 nm.

### Electron transmission microscopy

Isolated DEVs were fixed in PBS - 2% PFA and located on a grid cover with formvar/carbon during 20 min. Then, the grid was washed with PBS and fixed with 1% glutaraldehyde – PBS for 5 min. For inclusion and contrast the sample was stained with uranyl-oxalate for 5 min. Lastly, the grid was located over a drop with methyl-cellulose-uranyl-acetate in ice and allow to dry completely at RT. Samples were observed using a transmission electron microscope, JEOL JEM-1010 fitted with a Gatan Orius SC1000 (model 832) digital camera.

### Transwell assay

Immature BMDCs were cultured in the upper chamber of a transwell well over a 0.44 µm pore membrane (3413, Corning) and infected or not with *Lm* as previously described. Then, cells were incubated overnight in contact through the membrane with immature BMDCs seeded in the lower chamber. Cells in the lower chamber were collected for RT-qPCR analysis.

### Immunoblotting

Cells or EVs from BMDCs infected or not with *Lm* were lysed in western-blot lysis buffer (50mM Tris-HCl pH 8.0, 150mM NaCl, 1% Triton X-100, 0.1% sodium deoxycholate, and 0.1% SDS) supplemented with protease (Complete, Roche) and phosphatase (PhosSTOP, Roche) inhibitor cocktails. Cell lysates were cleared of nuclei by centrifugation at 15,000 × g for 15 min. Proteins were separated by 8-12% acrylamide/bisacrilamide gels and transferred to nitrocellulose or methanol-pre-treated PVDF membranes (Bio-Rad). Proteins were visualized with LAS-3000 (GE) or iBright 1500 (ThermoFisher Scientific) after membrane incubation with the corresponding primary antibodies and horseradish peroxidase (HRP)-conjugated secondary antibodies (1:5000) (**Table S1**).

### RNA extraction and real-time quantitative PCR (qPCR)

RNA was extracted from BMDCs using TRI-Reagent Solution (Ambion) following manufacturer instructions. Total RNA (2 µg) was retrotranscribed to cDNA using RT High Capacity cDNA Reverse Transcription Kit (Applied Biosystems) and amplified by qPCR using GoTaq^®^ qPCR Master Mix (Promega) by SYBR Green detection in an AB7900-384 termocycler (Applied Biosystems). Expression levels were normalized to *Ywhaz* and *β-Actin* housekeeping genes. Primers used are listed in **Table S2**.

### Flow cytometry (FC)

For FC analysis, DCs were harvested and suspended in PBS-1% BSA-5mM EDTA (PBE) with Fc-block (CD16/CD32, BD), washed and incubated with the corresponding fluorescence conjugated-primary antibodies in PBE. Singlet cells were discerned based on FSC and SSC parameters (pulse width and height). LIVE/DEAD^®^ Fixable Yellow Dead Cell Stain (L34968, Invitrogen) was used as a viability marker in PBS-5mM EDTA. Cells were then analyzed by FC in a BD FACS Canto II Cell Analyzer (BD). Data were analyzed with FlowJo v10 Software (BD).

### Enzyme-linked immunosorbent assay (ELISA)

DCs were cultured treated or not with DEVs overnight, supernatant was collected and frozen at -80°C for ELISA analysis. IL-1β assays were performed following the manufacturer instructions with undiluted DCs supernatant (88-7013-88, Invitrogen). Whole cell lysates and DEVs from HDAC6-WT and KO, infected or not with *Lm* were analyzed by LumiKine™ Xpress mIFN-β 2.0 (InvivoGen, luex-mifnbv2a) following manufacture instructions.

### Vaccinia virus infection

To assess the antiviral protection of DCs treated with DEVs, BMDCs were infected with recombinant Vaccinia Virus (VACV)-GFP (1:1) as described previously in (20).

### Protein extraction and tryptic digestion

The cell pellets were resuspended in a lysis buffer containing 50 mM Tris-HCl pH 6.8, 4% sodium dodecyl sulfate (SDS) and 10 mM dithiothreitol (DTT) and boiled for 5 min at 95 °C. Protein extracts (250 μg) were digested overnight at 37°C in 50 mM ammonium bicarbonate pH 8.8 with sequencing grade trypsin (Promega, Madison, WI, USA) at 1:40 (w/w) trypsin:protein ratio, using filter-assisted sample preparation technology (FASP, Expedeon, San Diego, CA, USA) (23). The EVs preparations were boiled at 95° for 5 min in the dark in the presence of a lysis buffer containing 50 mM Tris-HCl pH 8.8, 1% SDS and 50 mM iodoacetamide, after which reversibly oxidized protein thiol groups were reduced with DTT and then alkylated using S-methyl methanethiosulfonate, as published (24). Protein extracts (100 μg) were digested with trypsin under the conditions described above for the DCs. The resulting peptides were desalted on C18 Oasis HLB extraction cartridges (Waters Corporation, Milford, MA, USA) and dried-down.

### Peptide labeling and fractionation

Peptide samples from DCs and EVs were labeled with 8-plex and 4-plex isobaric tags for relative quantitation (iTRAQ, Sigma-Aldrich), respectively, according to the manufacturer’s instructions. The individually labeled samples were mixed appropriately, desalted using C18 Oasis HLB extraction cartridges (Waters) and dried-down. Peptides from DCs were taken up in 0.1% TFA and separated into five fractions by high pH fractionation (25). The labeled peptide samples from EVs were taken up in 5 mM ammonium formate pH 3 with 15% acetonitrile (AcN, Sigma-Aldrich) and separated into six fractions by mixed-mode, strong cation-exchange, reversed-phase (26) fractionation on Oasis MCX cartridges (Waters).

### Immunoaffinity enrichment of DC acetylated peptides

The iTRAQ-labeled peptide samples obtained from DCs were taken up in immunoaffinity purification buffer (IPA buffer: 50 mM MOPS/NaOH pH 7.2, 10 mM Na_2_HPO_4_, 50 mM NaCl), sonicated and incubated with anti-K-Ac kit (PTMScan, Pilot Acetyl-Lysine Motif [Ac-K] Kit, CST #14499). Flow-through and eluted samples (the latter enriched in acetylated peptides) were desalted using C18 Oasis HLB extraction cartridges (Waters) and ZipTip pipette tips (Millipore), respectively, and dried-down.

### Liquid chromatography mass spectrometry (LC-MS/MS)

DC samples were analyzed on an Easy nLC 1000 nano HPLC system coupled to a Q Exactive HF mass spectrometer (Thermo Scientific, San José, USA). C18-based reverse phase separation was used with a 50-cm analytical column (EASY-Spray, Thermo Fisher Scientific). Peptides were loaded in buffer A (0.1% formic acid (v/v)) and eluted with a 300-min linear gradient of 8-31% buffer B (90% AcN, 0.1% formic acid (v/v)) at 200 nL/min flow. Spectra were acquired using full ion-scan mode (120,000 FT-resolution) over the 400-1,500 mass-to-charge (m/z) range. Data acquisition was performed using a top 15 method (15,000 resolution). EV samples were analyzed on an Easy nLC 1000 nano-HPLC apparatus coupled to an Orbitrap Elite mass spectrometer (Thermo Scientific, San José, USA). Peptides were loaded onto a home-made C-18 reversed-phase nano-column (100 μm I.D., 45 cm) and separated in a continuous gradient consisting of 8-31% B for 180 min at 300 nL/min. Peptides were ionized using a Picotip emitter nanospray needle (New Objective, Woburn, MA, USA). Each MS run consisted of enhanced FT-resolution spectra (120,000 resolution) in the 390–1,200 m/z range followed by data-dependent MS/MS spectra of the 10 most intense parent ions (30,000 resolution) acquired along the chromatographic run.

### Peptide identification

DC LC-MS/MS data were analyzed with Proteome Discoverer 2.1 using SEQUEST-HT (Thermo Fisher Scientific), against a Uniprot database containing all sequences from *Mus musculus* and *Listeria monocytogenes* (January 2018; 93,699 entries). EV LC-MS/MS data were analyzed with Proteome Discoverer 1.4 (Thermo Scientific) using SEQUEST (Thermo Scientific) against a Uniprot database containing all sequences from *Mus musculus* and *Listeria monocytogenes* (May 2014; 72,435 entries). Database search parameters were selected as follows: trypsin digestion with four maximum missed cleavage sites, precursor mass tolerance of 800 ppm, fragment mass tolerance of 0.02 Da. Additionally, for DCs, Met oxidation, Lys acetylation, Lys ubiquitination and Lys iTRAQ 8-plex modification (+304.2054 Da) were considered as variable modifications, while Cys carbamidomethylation and peptide N-terminal modification of +304.2054 were set as fixed modifications. In the case of EVs, Cys carbamidomethylation, Cys methylthiolation, Met oxidation, Lys acetylation and Lys ubiquitination were considered as dynamic modifications. The results were analyzed using the probability ratio method (27), and a false discovery rate (FDR) for peptide identification was calculated based on the search results against a decoy database using the refined method (28) restricting tolerance to 15 ppm (29). A 1% global FDR was taken as a threshold for peptide identification. A local FDR was calculated for acetylated peptides, for which a 1% local FDR threshold was considered. Peptides were ascribed to the best protein proposed by the Proteome Discoverer algorithm.

### Peptide and protein quantification

The quantitative information from iTRAQ reporter intensities was integrated from the spectrum level to the peptide level and then to the protein level on the basis of the WSPP model (30) using the Generic Integration Algorithm (GIA) (31) under the SanXoT software package (32). This model provides a standardized variable, Z_q_, defined as the mean-corrected log2-ratio expressed in units of standard deviation at the protein level. Quantitative peptide values (Z_pq_) are referred to the weighted averages of the non-modified peptides from the same protein and therefore are not affected by protein abundance changes. This statistical model describes accurately the error distribution of abundance changes of both non-modified and modified peptides in null hypothesis experiments (33). Outliers at the peptide and protein levels were detected at 1% FDR as described in (30).

### Ingenuity Pathway Analysis (IPA)

For both the WT-*Lm* vs WT and the KO-*Lm* vs KO comparison, IPA (https://www.qiagenbioinformatics.com) was used with log_2_ fold-change (log2-FC) values fulfilling FDRq < 0.05 in, which were considered statistically significant changes.

### Statistical analysis

Data were analyzed with GraphPad Prism 8 software (La Jolla, CA), expressed as the mean ± standard deviation (SD) or Tukey box and whisker plot. Data that follow a normal distribution (test) were analyzed by Student’s t-test; Mann-Whitney U test was used for nonparametric data. Samples with more than two groups were analyzed with one-way analysis of variance (ANOVA) with a Tukey post-test for parametric data or Krustal-Wallis with Dunn’s post-test for nonparametric. Differences were considered significant when *P < 0.05, **P < 0.01, ***P < 0.001, ****P < 0.0001 and ns for not significant data.

## Results

### Differential protein repertoire of DCs and their derived EVs upon infection with *Listeria monocytogenes*

**Figure 1:**
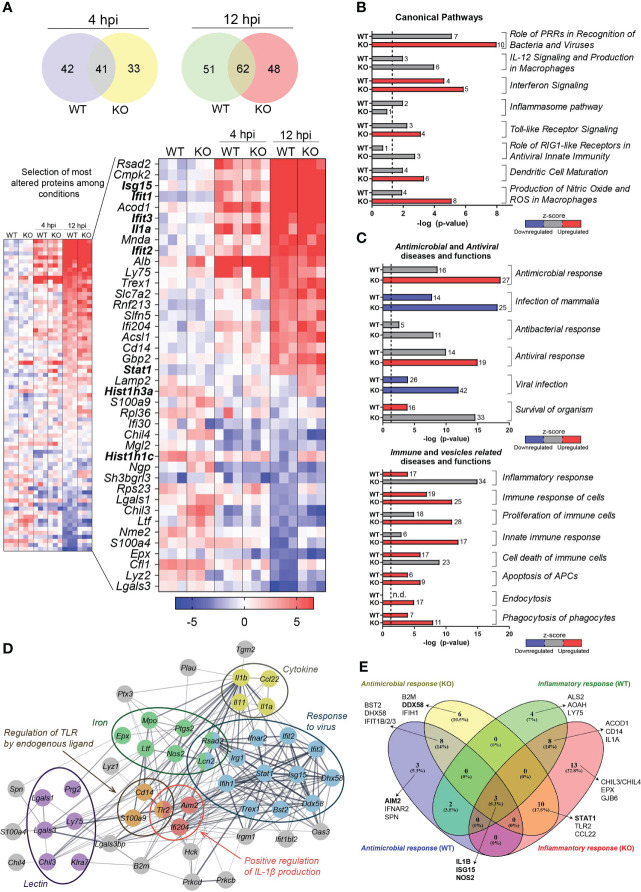
Proteome alterations in whole DC lysates after *Lm* infection. **(A)** Multiplexed proteomics analysis of *Lm-*infected or uninfected BMDCs from *Hdac6^+/+^* (WT) or *Hdac6^-/-^* (KO) mice (n = 3). Venn diagrams show the number of significant protein abundance changes detected in BMDCs after 4 h or 12 h of *Lm* infection (FDRq < 0.05 in at least one condition). The smaller heatmap shows quantitative results for those proteins with |Zq| > 2. The larger heatmap shows the top 20 up- and down-regulated proteins (the gene name is indicated) during *Lm* infection. **(B, C)** Canonical signaling pathways **(B)** and diseases and functions **(C)** predicted as altered 12 hpi by IPA. A –log (*P*-value) = 1.3 (*P*-value = 0.05) threshold was considered for statistical significance (dotted line). Four diseases and functions categories were considered: *antimicrobial*, *antiviral*, *immune* and *vesicles related* diseases and functions. Predicted upregulation (positive z-score, in red) or downregulation (negative z-score, in blue) is shown; z-scores corresponding to uncertain predictions are indicated in grey. n.d, non-detected. Bar numbers represent the number of protein components detected in the corresponding category. **(D)** Interaction network obtained with the proteins making up the *Antimicrobial response* and *Inflammatory response* categories shown in **(C)**. This set of proteins was found significantly enriched in several biological processes (*response to virus* (GO: 0009615), FDR=7.6 · 10^-13^; *positive regulation of interleukin-1 beta production* (GO: 0032731), FDR= 0.0141) and *regulation of TLR by endogenous ligand* (mmu5686938 KEGG pathway), FDR= 0.0043) and Uniprot annotations (*lectin* (KW-0430), FDR= 0.00026; *iron* (KW-0408), FDR= 0.00024; and *cytokine* (KW-0202), FDR= 0.0321). Proteins are labeled according to gene name. **(E)** Venn diagram represents a comparison of protein components involved in two specific predicted functions: antimicrobial response and inflammatory response, taking into account *Hdac6* ^+/+^ (WT) and *Hdac6* ^-/-^(KO) conditions.

To identify specific DC constituents triggered by *Lm* infection involved in innate immune processes and the potential role of HDAC6 we assessed the proteome alterations that took place in *Hdac6^+/+^* (WT) and *Hdac6^-/-^* (KO) BMDCs after *Lm* infection for 4 or 12 h in a multiplexed proteomics experiment. A total of 7137 proteins could be quantified upon LC-MS/MS. When comparing non-infected vs infected conditions, we found 113 and 110 to be altered in the WT and in the KO BMDCs, respectively (**Table S3** and **Figure 1A**). Significant abundance changes were already observed at 4h, but were amplified at 12h. We observed an increase in the abundance of antiviral proteins, e.g. Interferon stimulated gene 15 (ISG15), Interferon-induced protein with tetratricopeptide repeats 1 (IFIT1), IFIT3 or Signal transducer and activator of transcription 1 (STAT1) both in WT and KO BMDCs (**Figure 1A**), in line with what expected from a *Lm* infection (34). Interestingly, we observed a reduction of some proteins after infection at 12 h compared to non-infected cells in WT vs. KO cells, including histones such as HIST1H1C and HIST1H3A (**Figure 1A**).

IPA analysis revealed that *Lm* infection in KO BMDCs increased IFN signaling, pattern recognition receptors (PRRs) for bacteria and viruses, TLRs signaling, DC maturation, production of nitric oxide (NO) and reactive oxygen species (ROS). In WT BMDCs, alterations were related to PRRs, the inflammasome and IFN signaling (**Table S4** and **Figure 1B**). More generally, IPA analysis also indicated that 188 diseases and functions were altered in KO vs. WT-BMDCs (**Table S4**). Most notably, the analysis predicted alterations to immune processes (antimicrobial and antiviral responses, innate immune function), apoptosis and vesicle-related processes (**Figure 1C**).

IPA revealed a set of proteins shared by various related functional groups. We thus compared proteins across the most significantly changed groups (“*Antimicrobial Response*” and “*Inflammatory Response*”, see **Figure 1C**). These proteins are related with functions in antiviral responses, pathogen recognition, cytokines, iron-related processes or proteins from the lectin family, which are among the most significantly affected upon infection (**Figures 1A, D**). Interestingly, some of these proteins have a different behavior under the two conditions examined. For example, Absent in melanoma 2 (AIM2), which is part of one of the inflammasome complexes and DNA sensor for *Lm* (35), was altered only in WT BMDCs upon infection (**Figure 1E**). In addition, DExD/H-Box Helicase 58 (DDX58, also known as Retinoic acid-inducible gene I (RIG-I)), and STAT1, which is a key component of bacterial and viral sensing pathways, were only statistically altered during infection in KO DCs (**Figure 1E**). However, other proteins such as ISG15, Nitric Oxide Synthase 2 (NOS2), IL1-β and IFIT1 were similarly altered in both conditions upon infection (**Figure 1E**).

**Figure 2:**
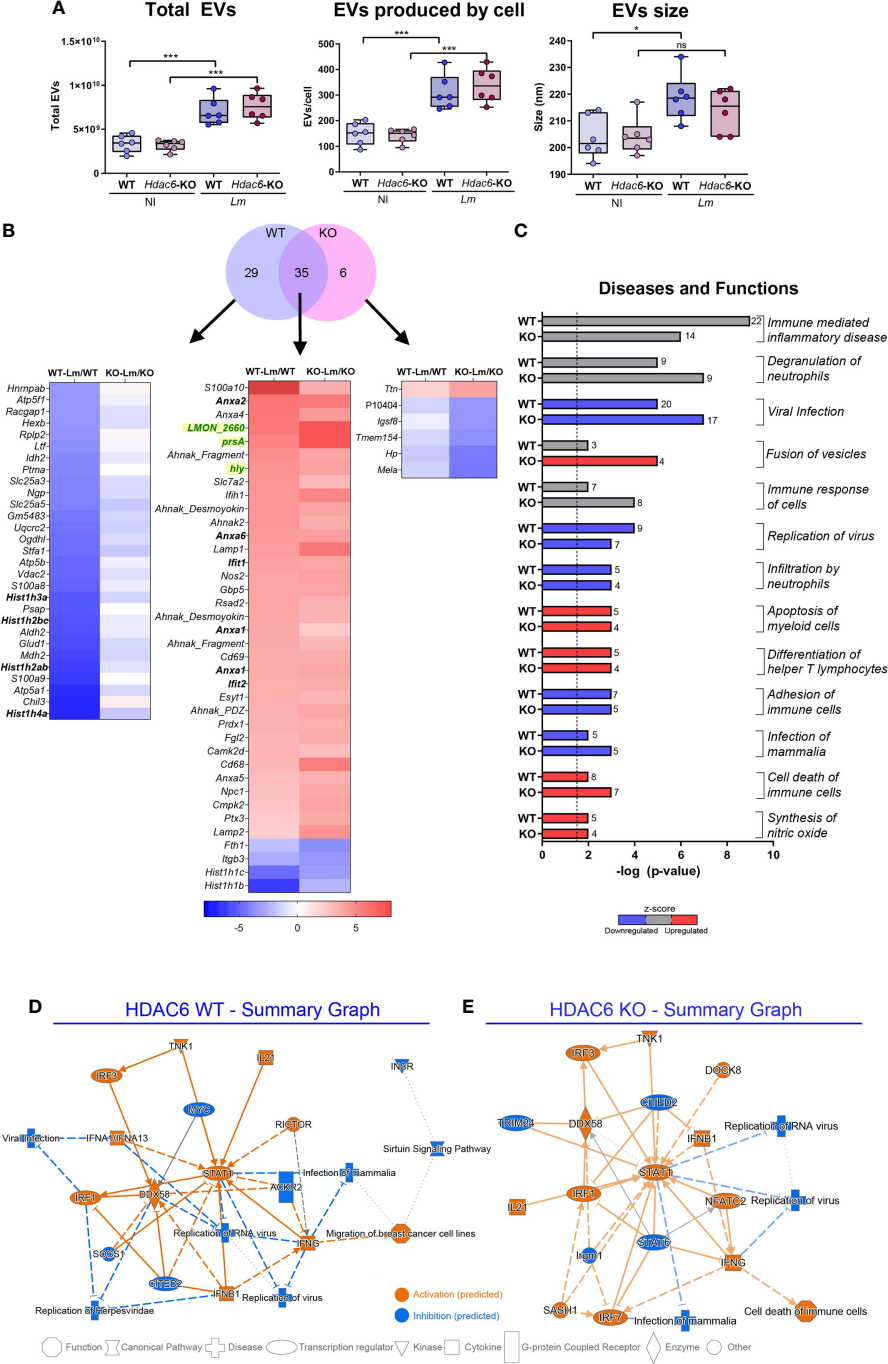
EVs secreted during infection with *Lm* present a different protein profile. **(A)** NTA of EVs isolated by ultracentrifugation from *Lm*-infected or non-infected *Hdac6^+^* ^/+^ (WT) or *Hdac6* ^-/-^ (KO) DCs. Plots show total EV numbers, number of EVs produced per cell and the mean size of the EV population in µm from left to right, respectively (*n* = 6, t-test; *P < 0.05, ***P < 0.001 and ns, not significant). Rows are labeled according to gene name. **(B)** Venn diagram shows the number of significant protein abundance changes detected in EVs of DCs after *Lm* infection (FDRq < 0.05 in at least one condition). The heatmap represents proteins altered in EVs for each group and condition (the gene name is indicated except for P10404, which lacks gene ascription). *Lm* proteins are colored in green. **(C)** Horizontal bar chart of diseases and functions predicted to be altered by IPA. A –log (*P-*value) = 1.3 (*P-*value = 0.05) threshold was considered for statistical significance (dotted line). Predicted upregulation (positive z-score, in red), or downregulation (negative z-score, in blue) is shown (z-scores corresponding to uncertain predictions are indicated in grey). Bar numbers represent the number of protein components detected in the corresponding category. **(D, E)** IPA graphical summary of WT **(D)** and KO BMDCs **(E)** upon 12 hpi presenting the key results of the analysis as a network. Solid lines depict direct interaction, slashed lines show indirect interaction and dotted lines represent inferred correlation from machine-based learning. Colors show IPA predictions (orange, predicted activation; blue, predicted inhibition; and grey, no prediction). Color intensity depicts grade of confidence.

Based on the crucial importance of EVs in immune cell-cell communication and the maturation of DCs against subsequent infection (20, 36), we next assessed whether EV production and secretion was altered during *Lm* infection in WT or KO BMDCs. Infection with *Lm* induced an increase in EV secretion (total and secreted number of EVs per cell) in both WT and KO BMDCs (**Figure 2A**). A significant increase in vesicle mean size in the WT DCs was observed upon intracellular bacterial infection (**Figure 2A** and **Figure S1A**). These results suggest a possible role of DEVs as a component of cellular crosstalk during infection (37). However, no significant differences were detected when DEV features from both genotypes were compared to each other (**Figure 2A**), suggesting that the absence of HDAC6 does not have an effect on EV number and size. Likewise, we have characterized the vesicles topology including electron microscopy micrographs (**Figure S1B**). Overall, with these data we confirm that the isolated vesicles present a characteristic cup shape and that no other contaminant is present.

Next, we studied the global changes in the repertoire of EV-loaded proteins during *Lm* infection (**Table S5**). A total of 35 proteins displayed significant changes in both conditions, 29 corresponding to the WT and 6 to the KO condition (FDRq < 0.05; ≥ 2 peptides identified). Notably, several members from Annexin A family (ANXA1, ANXA2, ANXA4, ANXA5 and ANXA6) were overrepresented in DEVs from *Lm*-infected BMDCs (**Figure 2B**) but remained unaltered in total cell lysates, suggesting a specific sorting during infection of these ANXA family members to EVs (**Figure S2A**). In addition, several histones (HIST1H3A, HIST1H2BC, HIST1H2AB, or HIST1H4A) were underrepresented in DEVs from WT BMDCs (**Figure 2B**), but modestly overrepresented in DEVs from KO cells (**Figure S2A**). Interestingly, *Lm* proteins were present in both conditions after infection (**Figure 2B**), suggesting that bacterial proteins are loaded in DEVs and may have a role in EVs-mediated communication during infection. One example is PrsA, a *Lm* foldase (*Lm*UniProtKB - Q8Y759, PRSA1_LISMO*LM*), which is a putative pheromone lipoprotein. Another example is *hly*, which is the gene encoding the pore-forming protein LLO (UniProtKB - P13128, TACY_LISMO) (3). We found significant changes during *Lm* infection in functions such as viral infection and virus replication, which were predicted by IPA analysis to be decreased in DEVs from infected BMDCs (**Figure 2C**). Conversely, differentiation of T helper cells was predicted to be increased (**Figure 2C**). **Figures 2D, E** depict IPA of DEVs from WT (**Figure 2D**) and KO (**Figure 2E**) BMDCs after *Lm* infection. Both genotypes share many common altered proteins, including several important mediators of antiviral and immunological responses (**Figures 2D, E**). However, the predicted activation status in the KO condition has lower statistical confidence (**Figure 2E**). STAT1 and DDX58 seem to be central elements during the cellular response to infection in whole cells as well as in their DEVs. IPA analysis returned a total of 245 diseases and functions altered in both conditions during infection (*P*-value < 0.05) (**Table S6**). Together, these analyses predict a decrease of proteins involved in immune alert and response in DEVs from infected cells (**Figures 2C–E**, in blue in the first). In contrast, loading of proteins involved in vesicle fusion is predicted to be increased by *Lm* infection (**Figure 2C**, in red), consistent with the observed increase in DEVs release upon infection.

**Figure 3:**
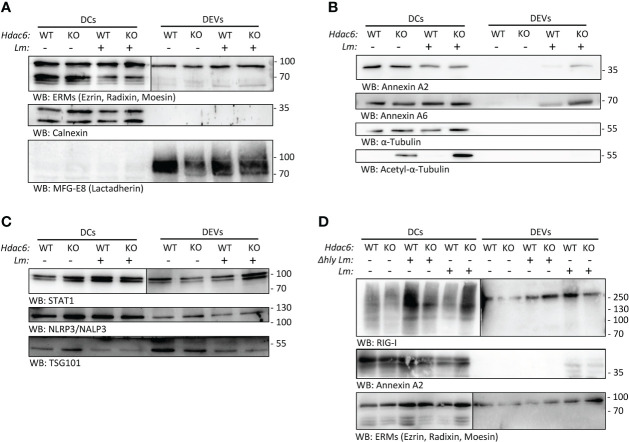
*Lm* infection skews protein content of BMDC-secreted EVs. **(A, B)** Western blot analysis of protein extracts prepared from total lysates from *Lm-*infected or non-infected *Hdac6* ^+/+^ (WT) or *Hdac6^-^* ^/-^ (KO) DCs and their respective secreted EVs (16 hpi). Cells and EVs were blotted for EV markers (ERMs and MFG-E8/Lactadherin) and an endoplasmic reticulum control (Calnexin) in A. Top right (DEVs, ERMs) is shown at a longer exposure time. In B, an analysis of Annexin A2 Annexin A6, Tubulin and acetylated tubulin is included being the sample the sample the same as in A. **(C)** Western blot analysis of STAT1, NLRP3/NALP3 and the EV marker TSG101 in DEVs samples from WT and KO-BMDCs infected or not with *Lm*. DEVs panel for STAT1 (on the right) is shown at a longer exposure time. **(D)** Western blot analysis of cells and EVs blotted RIG-I/DDX58, Annexin A2 and exosomal marker ERM (ezrin/radixin/moesin). In this experiment a Δ*hly Lm* strain-infected (Δ*hly*) condition was included. Right DEVs panel for RIG-I and ERMs is shown at a longer exposure time. Images are representative of one out of 3 independent experiments.

We characterized and validated by Western blot some of the proteins found regulated in the proteomics analysis. After infection, we detected EV markers such as Ezrin, Radixin and Moesin (ERMs), and other EV markers like Milk Fat Globule-EGF Factor 8 (MFG-E8, Lactadherin), but no contamination from other membrane compartments like the endoplasmic reticulum (CALNEXIN) (**Figure 3A**). Some members of the Annexin family found in both whole cell lysates and DEVs proteomics analysis, such as ANXA2 and ANXA6, were only present in DEVs after *Lm* infection (**Figure 3B** and **Figure S2B**). Interestingly, STAT1 was increased during infection in DEVs from KO BMDCs (**Figure 3C**). We observed no effect on the expression of NLRP3, a key inflammasome component (**Figure 3C**).

To study the contribution of the immune evasion ability of *Lm* to EV loading, we infected BMDC with a *hly*-deficient *Lm* strain (Δ*hly*). This strain cannot form the LLO pore and is thus unable to evade the endolysosome pathway (3). We observed an increased expression of RIG-I (DDX58) in whole cell lysates of KO DCs during wild type *Lm* infection (**Figure 3D**), but not during *hly*-deficient *Lm* infection. In addition, ANXA2 was not detected in EVs from BMDCs infected with *hly*-deficient *Lm* (**Figure 3D**). This indicates that *hly* and subsequent immune evasion crucially determines protein loading into EVs upon infection. Conversely, when looking at DEVs, RIG-I was observed in EVs derived from WT DCs, in agreement with proteomics data (**Figure S2B**).

### DEVs proteins display specific post-translational modifications (PTMs)

**Figure 4:**
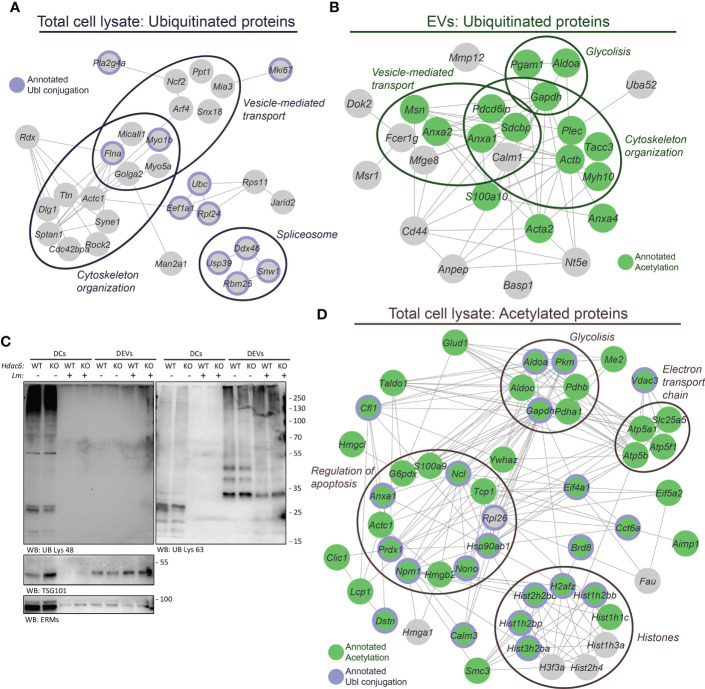
Post-translational modifications play a role in infection and sorting to EVs. **(A)** The 41 ubiquitinated Lys sites identified in the DC lysates pertain to a set of proteins found significantly enriched in the following biological processes: *cytoskeleton organization* (GO:0007010), FDR= 0.00036; *vesicle-mediated transport* (GO:0016192), FDR= 0.0075; and *spliceosome* (mmu03040 KEGG pathway), FDR=0.0278. In addition, 11 of these proteins were annotated as *Ubl conjugation* (KW-0832, FDR= 0.0040) in the Uniprot database. **(B)** Analysis of the 49 ubiquitinated Lys sites identified in the EVs revealed a different profile of ubiquitinated proteins as compared to the DC lysates. However, functional enrichment analysis showed an overrepresentation of proteins related to *cytoskeleton organization* (GO:0007010), FDR= 0.0119; *regulation of vesicle-mediated transport* (GO:0060627), FDR= 0.0037 and *glycolytic process* (GO:0006096), FDR= 0.0188. Fifteen of these ubiquitinated proteins were previously annotated under *Acetylation* (KW-0007, FDR= 5.8 · 10^-5^) in the UniProt database. **(C)** Western blot analysis of protein ubiquitination. Protein extracts were prepared from EVs and *Hdac6* ^+/+^ (WT) or *Hdac6* ^-/-^ (KO) DCs samples infected or not with *Lm*. Cells and EVs were blotted with an antibody against polyubiquitin chains linked through Lys48 ubiquitin (UB) residue (left panel) or Lys63 UB residue (right panel). TSG101 and ERMs were used as EV markers. Both panels correspond to same sample. **(D)** The 60 proteins harboring the 79 acetylated Lys sites identified in the DC lysates showed an overrepresentation of proteins implicated in *chromatin organization* (GO:0006325), FDR= 0.00032; *regulation of apoptotic process* (GO:0042981), FDR= 0.0205; *glycolytic process* (GO:0006096), FDR= 0.0014; and *electron transport chain* (WP295 WikiPathway), FDR= 0.0151. Out of these 60 acetylated proteins, 44 and 22 proteins were found annotated as *Acetylation* (KW-0007, FDR= 6.6 · 10^-25^) and *Ubl conjugation* (KW-0832, FDR= 1.5 · 10^-6^) in the UniProt database, respectively. The proteins annotated as *Acetylation* are indicated in green and proteins annotated as *Ubl conjugation* are denoted by a purple edge. Proteins are labeled according to gene name.

Diverse PTMs (acetylation, ubiquitination, SUMOylation and others) control protein sorting into EVs (15, 16). Following this regulation, we assessed PTMs in our samples. Functional analysis of the set of BMDC proteins found increased (Z_q_ > 2) upon *Lm* infection, of which 52% were also quantitated in the DEV proteome, revealed an enrichment of both acetylated and ubiquitinated proteins (**Figure S3**). In fact, we identified 41 ubiquitinated peptides in the proteome of BMDCs (**Table S7**), and some of these peptides belonged to proteins known to be ubiquitinated, e.g. E3 ubiquitin-protein ligase RNF213 (RNF213) and Polyubiquitin-C (UBC). Functional enrichment analysis of these ubiquitinated proteins showed a significant overrepresentation of *cytoskeleton organization*, *vesicle-mediated transport* and *spliceosome* (**Figure 4A**, **Table S8**). On the other hand, in the DEV proteome we found 49 ubiquitinated peptides from 35 different proteins, none of which had been previously annotated as ubiquitinated. However, several of these proteins were annotated as acetylated according to the Uniprot database. Despite that the ubiquitination profile detected in DEVs was completely different from that found in whole cell lysates, functional enrichment analysis suggested that “*cytoskeleton organization*” and “*vesicle-mediated transport*”, two functional categories found altered in the ubiquitinated DC proteins, were also significantly overrepresented in DEVs (**Figure 4B**, **Table S8**).

Based on a previous study that detected and differentiated between different ubiquitin topologies in urine human samples (38), we next evaluated protein ubiquitination in DEVs during the infective process by Western blot. Interestingly, Lys48-linked poly-ubiquitin (K48-polyUb) chains were found in cell lysates, whereas, Lys63-polyUb chains were mainly detected in EVs (**Figure 4C**). This observation could indicate the existence of an active sorting mechanism for EV-proteins encoded by different ubiquitination topologies. Likewise, similar results on the differential expression of K48-polyUb/K63-polyUb were obtained using EVs from T lymphocytes (**Figure S4**).

In addition, we carried out an immunoaffinity-based enrichment for acetylated peptides in the BMDCs lysates to ascertain the link between acetylation and the absence or presence of HDAC6 or the infection process. This enrichment step yielded 79 acetylated peptides (local FDR < 0.01) that belonged to 60 proteins, of which 44 and 22 had been previously annotated as acetylated and ubiquitinated proteins, respectively (**Figure 4D**). As expected, the most abundant acetylated proteins were histones, with 22 acetylated Lys residues. We also found an overrepresentation of proteins implicated in glycolysis, respiratory electron transport chain and regulation of apoptosis (**Figure 4D**, **Table S8**). However, in the DEVs only 18 acetylated peptides were identified (local FDR < 0.01), due to the limited amount of starting material (**Table S7**). Even so, functional enrichment analysis showed that some proteins related to the immune system were significantly overrepresented in this set of acetylated proteins, including C8B, ANPEP, IGF2R, and GRB2, among others (**Table S8**). Only one of the 18 acetylated peptides identified in DEVs (pertaining to cofilin-1) was also present in the acetylome from whole cell lysates.

Quantitative analysis of relative abundance changes at the peptide level revealed an alteration of the acetylation and ubiquitination profile upon *Lm* infection, both in whole BMDCs lysates and DEVs. However, no significant differences were observed between WT and KO (**Table S7**), suggesting that HDAC6 is not actively deacetylating targets during *Lm* infection. Alternatively, another deacetylase could be compensating the loss of HDAC6. Moreover, the lack of significant changes in the ubiquitination profile could indicate that the possible crosstalk between acetylation and ubiquitination coordinated by HDAC6 (39) is not critical in the *Lm* infection. While no significant changes were found in the acetylation profile between WT and KO conditions, the alterations unveiled at the protein abundance level suggest that HDAC6 may control protein abundance and/or EV loading independent of its deacetylating activity.

### DEVs from infected DCs induced antiviral genes and confer protective immunity to viral infection in recipient DC

**Figure 5:**
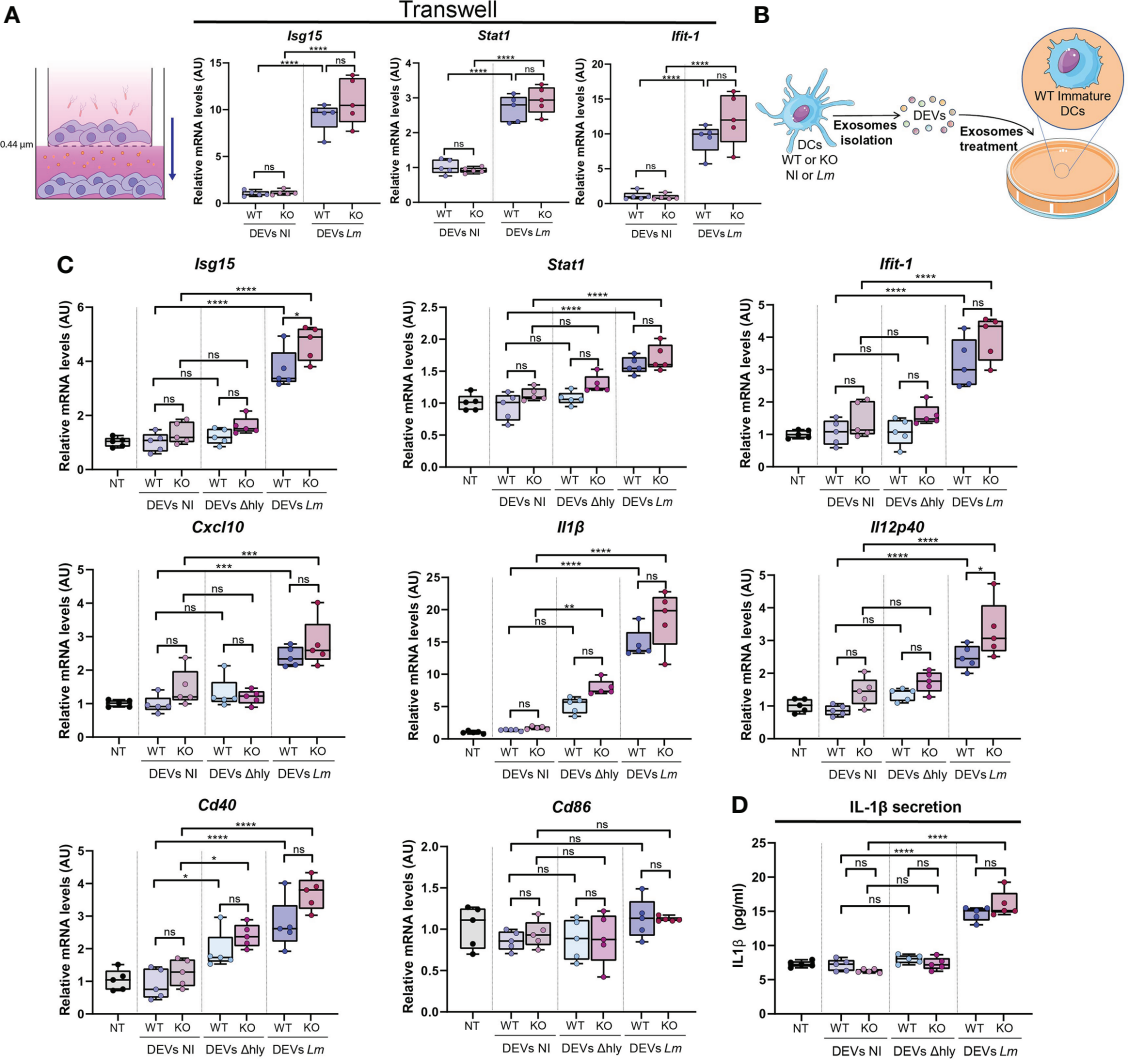
DEVs isolated from *Lm*-infected BMDCs induce gene expression of anti-pathogenic genes in immature BMDCs. **(A)** BMDCs were infected or not with *Lm* (upper chamber) and co-cultured in contact with other immature BMDCs (lower chamber) through a 0.4 µm pore membrane. qPCR analysis was performed in BMDCs cultured in the lower chamber for *Isg15, Stat1* and *Ifit-1* genes (*n=*5, one-way analysis of variance (ANOVA) test with Tukey’s posttest; ****P < 0.0001 and ns, not significant). Values were normalized with *Ywhaz* and *β-Actin* housekeeping genes. **(B)** Isolated DEVs by ultracentrifugation from *Lm*-infected (DEVs *Lm*) or non-infected (DEVs NI) *Hdac6* ^+/+^ (WT) or *Hdac6* ^-/-^ (KO) DCs were added to wild-type immature DCs for 24h. **(C)** qPCR analysis of the expression levels of *Isg15, Stat1, Ifit-1, Cxcl10, Il1β, Il12 p40, Cd40* and *Cd86* in DCs treated with vehicle (NT), isolated DEVs from non-infected DCs (NI), Δ*hly Lm* strain-infected (Δ*hly*) or *Lm*-infected (*Lm*) (*n=*5, one-way ANOVA test with Tukey’s posttest; *P < 0.05, ***P < 0.001, ****P < 0.0001 and ns, not significant). *Ywhaz* and *β-Actin* were used as housekeeping genes. **(D)** ELISA analysis in DEVs-treated DCs of IL-1β. Immature DCs were treated with DEVs from non-infected DCs (NI), Δ*hly Lm* strain-infected (Δ*hly*) or *Lm*-infected (*Lm*) (*n=*5, one-way ANOVA test with Tukey’s posttest; ****P < 0.0001 and ns, not significant).

DEVs exert diverse effects on an array of immune functions and cells, from antigen presentation to Natural Killer (NK) functions against tumors (18). The role of DEVs during intracellular bacterial infection has yet to be elucidated. We tested whether the observed differences in DEV protein content upon *Lm* infection could have a functional impact on the ability of DEV to alert the immune system. To do so, we treated immature wild-type BMDCs with DEVs from non-infected or infected BMDC. To prevent the presence of active bacteria, culture media of *Lm-*infected BMDC were filtered through 0.22 µm and 0.45 µm filters. No bacterial growth was observed after three days of culture (**Figure S5A**). Since EVs include vesicles 200-250 nm diameter (**Figure 2A**), we used 0.45 µm filters to prevent skewing the data based on the size of the filtered EVs. To rule out the effect of cell-cell contacts, we seeded BMDCs, infected or not with *Lm*, in the upper compartment of a 0.4 µm Transwell device. The bottom compartment was loaded with immature DCs. After 24h, we detected a significant induction of antiviral genes, e.g. *Isg15, Stat1* and *Ifit-1*, in immature DCs that have been cultured in contact with infected BMDCs (**Figure 5A**). In a separate set of experiments, we treated immature DCs with isolated DEVs from *Lm*-infected BMDCs for 24 h (**Figure 5B**). We observed a significant increase in the expression of anti-viral genes such as *Isg15, Stat1, Ifit-1* and *Cxcl10* (**Figure 5C**). Moreover, we found significant differences in cytokines such as *Il-1b* or *Il-12 p40* or DC maturation genes such as *Cd40*, but not *Cd86* (**Figure 5C**). Interestingly, EVs isolated from DCs infected with the Δ*hly* strain did not increase expression of these genes (**Figure 5C**). When isolated DEVs where filtered through 0.22 µm pore size, only *Isg15* expression was significantly increased (**Figure S5B**). This supports that these vesicles acquire a larger size after infection, so that when this filter is employed a significant proportion of vesicles will be restrained, which are at least partly responsible for the observed effect. Not unexpectedly, increased mRNA of *Il-1b* correlated with increased IL-1β cytokine secretion (**Figure 5D**). Moreover, IFN-β was detected in DEVs following *Lm* infection and with reduced expression in HDAC6-KO condition (**Figure S6**). However, this was not in accordance with the elevated ISG expression tendency in DCs treated with DEVs from HDAC6-KO. Besides, type I IFN, in order to signal through its surface receptor, would need to exit the vesicle or the cell after DEV internalization. Together, these results suggest that EVs secreted by DCs during *Lm* infection could prime immature DCs to acquire an “alert” state, which is likely useful to improve immune responses upon infection with virus and intracellular bacteria.

**Figure 6:**
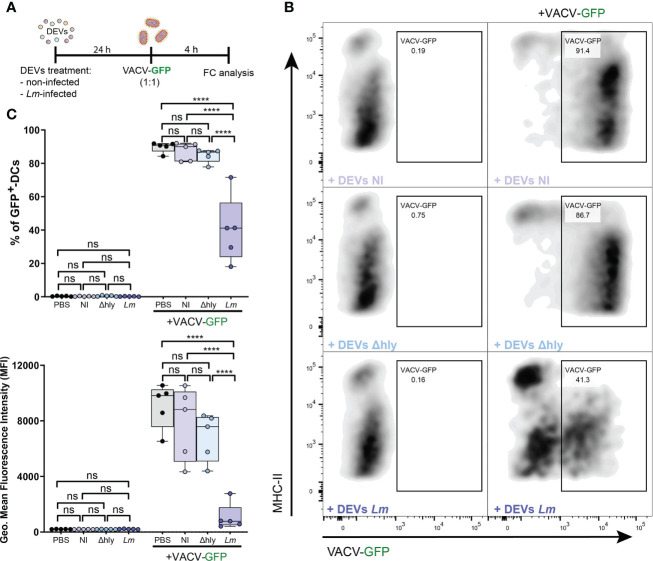
DEVs isolated from *Lm*-infected BMDCs protect against subsequent viral infections. **(A)** BMDCs were treated with PBS or DEVs from non-infected, Δ*hly-Lm* or *Lm*-infected BMDCs. After 24h, treated BMDCs were infected or not with recombinant VACV-GFP for 4 hours in 1:1 ratio. The level of infection was measured by flow cytometry as GFP^+^ infected cells. **(B)** Dot plots shows the gating strategy used to detect the level of BMDCs infection. **(C)** Statistical analysis of BMDCs infected with recombinant VACV-GFP. BMDC population was gated on alive CD11c^+^MHCII^+^ cells. (*n=*5, one-way ANOVA test with Tukey’s posttest; ****P < 0.0001 and ns, not significant).

As a proof of concept to ascertain whether the functional impact and potential anti-pathogenic response triggered by DEVs could induce protection against reinfection by a different pathogen, DCs were either treated or not with DEVs from non-infected or *Lm-*infected DCs and then were subjected to infection with a recombinant VACV-GFP. DEVs from *Lm*-infected DCs exerted more protection against VACV infection compared to DEVs from non-infected cells (**Figure 6**). This was observed in the reduction of the percentage of infected DCs (**Figures 6B, C**, *upper plot*) and the level of GFP expression (**Figure 6C**, *lower plot*), suggesting a reduction in viral replication or infection. In accordance with the gene expression data, DEVs from Δ*hly* strain infection do not confer this protection (**Figure 6**). This confirms that *hly* plays a crucial role in this process and that productive infection (as opposed to *Lm* ingress into host cells) is needed to the development of EV-mediated immune processes (40). Taken together, these data indicate that EVs derived from infected DCs induce an anti-pathogenic state that improves the response against subsequent viral infection with VACV in immature recipient DCs.

## Discussion

EVs are increasingly acknowledged as crucial elements to understand how cells communicate with each other either locally or systemically, with important consequences in cancer and the immune response. Recent studies have highlighted how EVs may prepare innate immune cells to respond to subsequent challenges by the same, or similar, pathogens, forming the basis of a form of “innate memory”. Here, we have focused on characterizing the content of EVs during *Lm*, and the potential role of the cytoplasmic deacetylase HDAC6 in EV cargo selection. The rationale for this choice was that HDAC6 is important in cytotoxic vesicle trafficking in CD8^+^ lymphocytes (41) and during *Lm* infection in DCs (8).

A first observation is that *Lm* infection increases EV formation in BMDCs independent of the expression of HDAC6. This is not unexpected and lends support to the hypothesis that DCs generate specific “alert” signals that prepare neighbor cells to respond to infection. This effect was independent of HDAC6 expression, which indicates that HDAC6 is not required for bacteria to increase EV production by host cells. However, some of the mechanisms altered in KO cells include antiviral and bacterial response signaling pathways. Moreover, HDAC6 inhibition increased the release of pro-inflammatory cytokines (TNF and IL-12) upon infection with other bacteria, e.g. *Mycobacterium tuberculosis* (42). Our data also show that annexins are heavily altered during *Lm* infection. ANXA2 ability to bind membrane phospholipid made it relevant to many membrane-related cellular functions (43). For instance, ANXA2, is necessary for Multivesicular Bodies (MVBs) biogenesis (44) and involved in vesicles formation during macroautophagy (45), thus having an important role in vesicle trafficking. Regarding inflammation, ANXA1 curbs leukocyte infiltration, activates neutrophil apoptosis and steers macrophage reprogramming toward a resolving phenotype (46). Other family member that has also been described as a component of the immune response is ANXA6 which is involved in the regulation of IL-2 and therefore T-cell proliferation (47). Taking all the above into account annexins are important players in endosome dynamics and formation; and in various immune mechanisms needed for the resolution of inflammation. Hence, the observed changes in expression could be part of the resolution of *Lm* infection. They can also be regulating the amount of secreted DEVs. These points merit further investigation.

*Lm* infection of BMDCs affected some proteins in the inflammasome pathway. This could be due to the fact that *Lm* infection is sensed by both NLRP3 and AIM2 inflammasome components (48). We confirmed this when we identified AIM2 as a protein altered only in WT cells. AIM2 is a sensor of *Lm* DNA and part of one of the inflammasome complexes that controls the release of the inflammatory cytokines IL-1β and IL-18, as well as pyroptotic cell death in *Lm*-infected cells (35). In this regard, AIM2 inflammasome is downregulated when another member of the HDAC family, HDAC3, is inhibited presumably because of enhanced STAT1 acetylation and subsequent attenuated STAT1 phosphorylation (35). This means that STAT1 acetylation is a regulatory hotspot that positions this pathway under the control of multiple HDAC proteins. STAT1 induces the expression of IFN-γ-induced genes, which can in turn be modulated by other HDAC members (49), that can be influenced by *Lm* as it could also modulate cytokine production in macrophages by increasing STAT1 phosphorylation (50). This is in agreement with our data showing that STAT1 is preferentially loaded into DEVs from KO BMDCs. In contrast, NLRP3 expression showed no significant changes. However, HDAC6 may only affect NLRP3 at a functional level by reducing its activation, but no its translation (51).

On the other hand, DDX58 (RIG-I) appeared altered during infection only in whole cell lysates from KO BMDCs, whereas it was mainly present in DEVs in WT cells. RIG-I is a cytosolic PRR that initiates the immune response against many RNA viruses. It can also detect *Lm* by sensing secreted bacterial nucleic acids (52). In addition, its activation is regulated by PTMs including deacetylation by HDAC6 (53, 54). The deficient targeting of RIG-I into KO DEVs despite being more abundant in whole cell lysates strongly suggests that HDAC6 participates in RIG-I sorting into EVs under *Lm* infection. Consequently, acetylation would constitute a retention signal that prevents EV targeting, at least for this protein. In agreement with this possibility, acetylation of high glucose-regulated protein 78 (GRP78) promoted protein retention in the cell, preventing its sorting into EVs in a colon cancer model (55).

Protein loading into EVs is tightly regulated by PTMs (15, 16). A specific example is the SUMOylation of hRNPA2B1, which affects its ability to load specific microRNAs into EVs (56). *Lm* infection inhibits SUMOylation of host proteins, which can be interpreted as a mechanism by which the infecting bacteria counter immune responses (57). This is further supported by the fact that specific PTMs decrease infectivity, e.g. ISGylation inhibits *Lm* infection (58). When analyzing protein PTMs in WT and KO cells, we observed that HDAC6 deletion did not affect the degree of global protein acetylation. This is consistent with HDAC6 having catalytic-independent roles, as we showed for T cell chemotaxis (59). HDAC6 possesses a zinc finger ubiquitin-binding motif (ZnF-UBP) as well as a SE14 domain that play fundamental roles in controlling tau aggregation (60). The ability of HDAC6 to control cytoskeleton dynamics and autophagy seems to be related to a deacetylase-independent scaffold function linked to the endosome-lysosome-phagosome pathway (8, 41, 61).

*Lm* infection also skews the pattern of host protein ubiquitination. Our data are consistent with the possibility that accumulation of proteins modified with K48 or K63 polyUb chains serves as an active EV sorting system: proteins modified with K63 chains are preferentially present in EVs, whereas proteins tagged with K48 chains are retained in the cell body. A preferential order of sorting of ubiquitin-modified proteins into EVs has been described in urine samples in agreement with the present findings. Accordingly, deubiquitination is dispensable for EV targeting (38).

Despite all the previous observations regarding the protein content between WT and KO-BMDCs, the main differences were observed when comparing infected vs non-infected conditions. Therefore, the absence of HDAC6 is important during *Lm* infection but does not seem to be essential for EV subsequent function to induce differentially an anti-viral gene program. However, in line with this, we have previously described that HDAC6 deficiency increases bacterial burden (8) and this might contribute to increased gene expression during *Lm* infection compared to WT DEVs as observed tendency in **Figure 5C**. Interestingly, these vesicles are larger after *Lm* infection which could be a result of the changes induced in the endosome-lysosome-phagosome pathway after a bacterial infection for its removal. This might translate into a more active mechanism of protein loading into vesicles and thus their increment in size. Nevertheless, to our knowledge differences in sizes might also be related to the origin of these EVs, which deserves further characterization, although typical exosomal proteins such as TSG101 and MFG-E8 were detected.

Our experiments revealed that *Lm* proteins are also sorted into host cell DEVs upon infection. Gram-positive bacteria secrete small-sized (20-100 nm) EVs that include an array of virulence factors (62, 63). Our data indicated that EVs secreted by BMDCs during *Lm* infection have an alerting effect on bystander, uninfected BMDCs, priming them against infection. One possibility is that infecting *Lm* secreted their own EVs, and their interaction with uninfected cells had that effect. Whereas our experiments do not entirely rule out this possibility, we minimized the chance of residual bacterial components affecting bystander cells by applying ultrafiltration, as described elsewhere (64, 65) and antibiotic treatment. Also, according to ISEV guidelines (66), the number of vesicle-producing cells was used as normalization for the EV dose in experiments to treat bystander DCs. However, future studies should consider incorporating NTA as a routine method for more complete characterization and better consistency of EV doses used. At any rate, the alerting effect was dependent on the infectivity of the *Lm* strain, as EVs of *Lm*-infected DCs activate immunity a strain deficient in LLO (Δ*hly*) and thus unable to escape the vacuole to be released into the cytoplasm did neither trigger antibacterial gene expression, nor the anti-pathogenic effect against reinfection in bystander BMDCs. Interestingly, LLO (*hly*) was found in BMDC EVs by MS. In any case, the loss of the effect when LLO is not present may suggest an important role of LLO-loaded EVs in alerting neighboring cells of the current infection to be further studied.

The anti-pathogenic effect observed in bystander cells included increased expression of genes such as *Isg15* and inflammatory cytokines such as *Il-1b.* These effects are similar to those observed when DCs form immune synapses with T cells, through which DCs receive instructions to express antiviral genes when the system is challenged by VACV infection (20). Interestingly, when DCs are treated with DEVs derived from infected DCs the results were similar. IL-1β and IL-12 are key pro-inflammatory cytokines important for the fighting against bacterial and viral infection (67). In this view, DEV release may constitute an autocrine-paracrine loop of immune alertness.

## Data availability statement

The datasets presented in this study can be found in online repositories. The names of the repository/repositories and accession number(s) can be found in the article/**Supplementary Material**. The mass spectrometry proteomics data have been deposited to the ProteomeXchange Consortium via the PRIDE (68) partner repository with the dataset identifier PXD033995 (whole cells lysates) and PXD033998 (EVs). Additional data related to this paper may be requested from the authors.

## Ethics statement

The animal study was reviewed and approved by the local Ethics Committee for Basic research at the CNIC Ethical Committee for Animal Welfare and the Órgano Encargado del Bienestar Animal (OEBA) del Gabinete Veterinario de la Universidad Autónoma de Madrid (UAM). This committee approved the document with an associated identification number PROEX 158/15 and PROEX 206.1/20.

## Author contributions

RI-S, IF-D, OM-G, and FS-M designed most experimentation and analyzed results; DC-F and MR-H helped with the collection of data and experimental design. EV helped with project design and provided *Lm* strains. EM-G participated in the bioinformatics analysis of the proteomic data and experimental design. MV-M helped with project design, critical reading and English editing of the manuscript. IJ, EC, and JV participated in the collection and analysis of the acetylome and proteomic data. JA participated in the collection of the acetylome data. RI-S and IF-D made the figure and wrote the manuscript with input from the rest of the authors. FS-M supervised and revised all the work. All authors contributed to the article and approved the submitted version.

## Funding

This study was supported by grant PDI-2020-120412RB-I00, PDC2021-121797-I00, BIO2015-67580-P and PGC2018-097019-B-I00 from the Spanish Ministry of Economy and Competitiveness (MINECO), grant S2017/BMD-3671-INFLAMUNE-CM from the Comunidad de Madrid, a grant from the Ramón Areces Foundation “Ciencias de la Vida y la Salud” (XIX Concurso-2018), “la Caixa” Banking Foundation (grants HR17-00016 and HR17-00247), BIOIMID (PIE13/041) and PRB3 (IPT17/0019 - ISCIII-SGEFI/ERDF, ProteoRed) from Instituto de Salud Carlos III, CIBER Cardiovascular (CB16/11/00272), and Fondo de Investigación Sanitaria del Instituto de Salud Carlos III and co-funding by Fondo Europeo de Desarrollo Regional FEDER). IF-D is supported by a Fellowship from the Spanish Ministry of Science, Innovation, and Universities (FPU15/02539). DC-F is supported by a Fellowship from “la Caixa” Foundation (LCF/BQ/DR19/11740010). The CNIC is supported by the Instituto de Salud Carlos III (ISCIII), the Ministerio de Ciencia e Innovación (MCIN) and the Pro CNIC Foundation, and is a Severo Ochoa Center of Excellence (CEX2020-001041-S). Funding agencies did not intervene in the design of the studies, with no copyright over the study.

## Acknowledgments

Authors thank Dr. Salvador Iborra for the help with recombinant VACV-GFP handling and preparation. We thank Cell cytometry, Proteomics, and Animal Facility Units from CNIC. We thank Francisco R. Urbano-Olmos and Covadonga Aguado-Ballano from the Transmission Electron Microscopy Laboratory at the Universidad Autónoma de Madrid (UAM).

## Conflict of interest

The authors declare that the research was conducted in the absence of any commercial or financial relationships that could be construed as a potential conflict of interest.

## Publisher’s note

All claims expressed in this article are solely those of the authors and do not necessarily represent those of their affiliated organizations, or those of the publisher, the editors and the reviewers. Any product that may be evaluated in this article, or claim that may be made by its manufacturer, is not guaranteed or endorsed by the publisher.
