# Supplementary material for: Extracellular vesicles from Listeria monocytogenes-infected dendritic cells alert the innate immune response
Source: Front Immunol. 2022 Sep 5;13:946358. doi: 10.3389/fimmu.2022.946358 (PMC9483171; doi:10.3389/fimmu.2022.946358)
Supplement: Supplementary file 1 [file DataSheet_1.pdf]

Supplementary Material

1 Supplementary Data

Fig. S1-6

Table S1-8

2 Supplementary Figures and Tables

2.1 Supplementary Figures

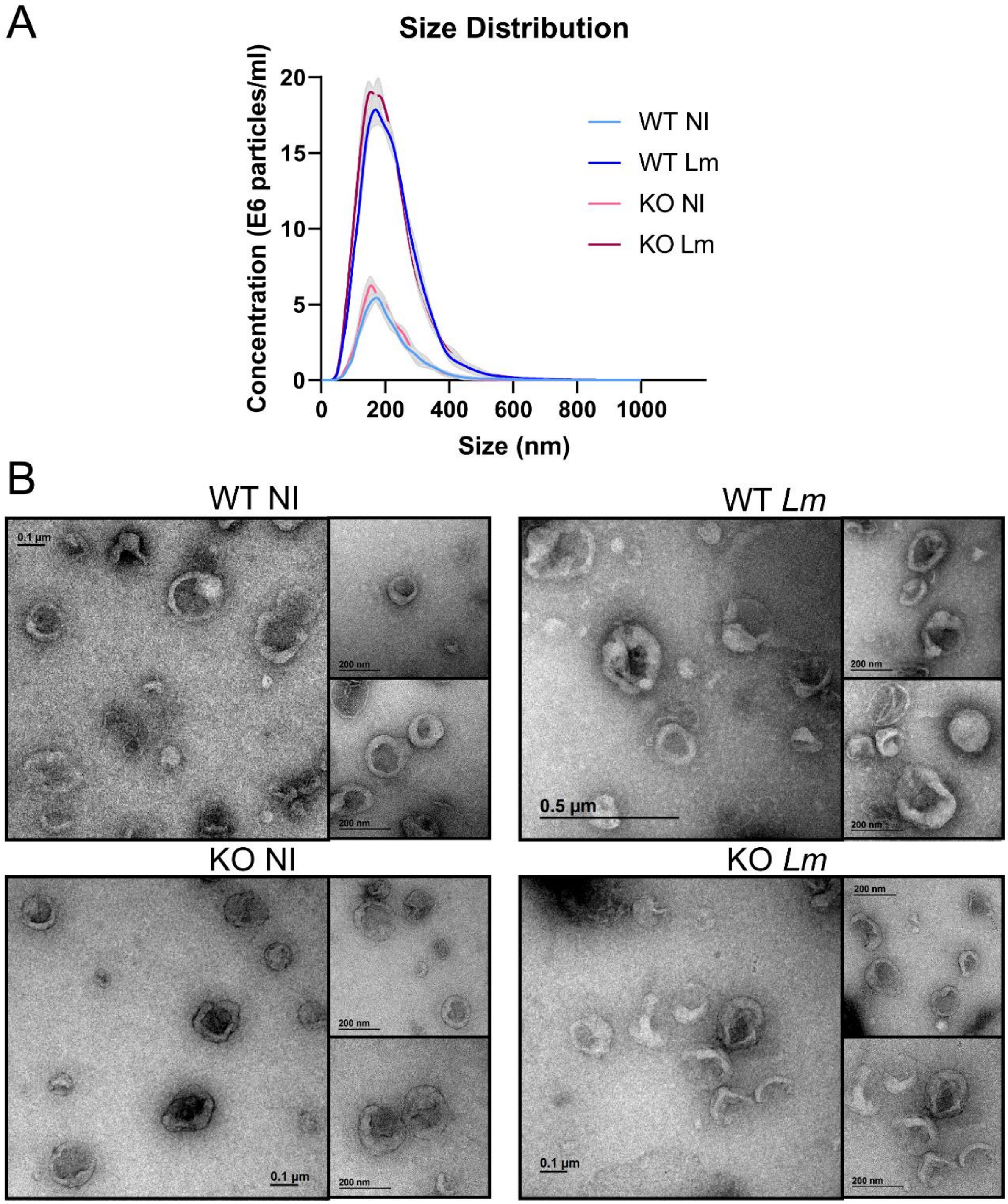

Supplementary Figure 1. Isolated EVs present typical size and topology. (A) NTA size profile distribution. Grey area indicates standard error of the mean. **(B)** Transmission electron microscopy images from DEVs isolated from non-infected (NI) or *Lm* infected DCs. For each condition, three images are shown. Magnification for the image in the left: × 80,000. Top right image magnification: x120,000. Bottom right image magnification: x150,000. Scale bar is included for each image (0.5µm, 0.1µm or 200nm).

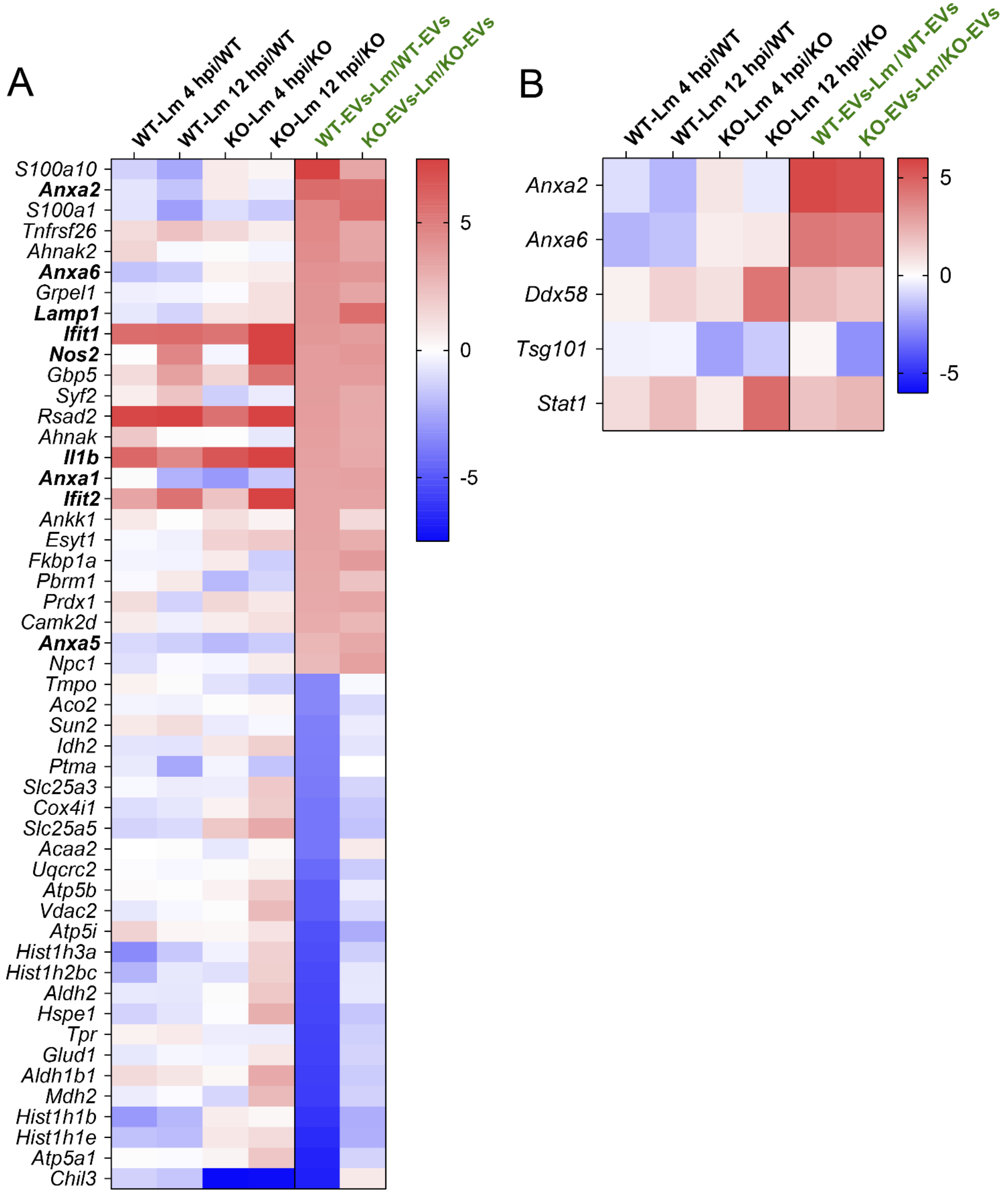

Supplementary Figure 2. Protein profiling from total cell lysates and their derived EVs from WT and KO-HDAC6 BMDCs. (A) Significant protein abundance changes shared between the total cell lysate and EVs after *Lm* infection (FDRq <0.05). **(B)** Protein abundance in total cell lysates and EVs observed in western-blot validation. Rows are labeled according to gene name.

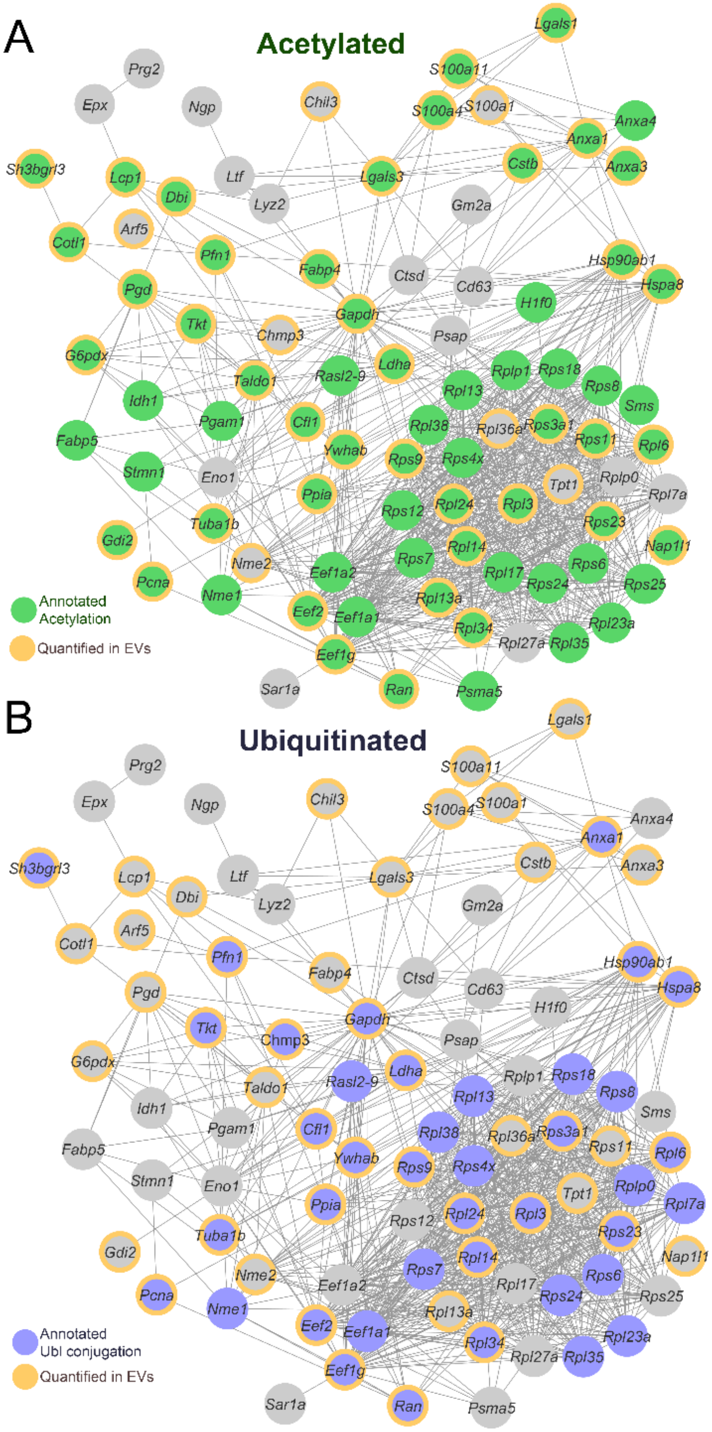

Supplementary Figure 3. Enrichment in acetylated and ubiquitinated DC proteins upon *Lm* infection. The 95 proteins identified with at least two peptides showing increased abundance (Zq > 2) in *Hdac6*$^{+/+}$ after 12 hours of *Lm* infection were submitted to STRING functional enrichment analysis. Results indicate significant overrepresentation of **(A),** acetylated (UniProt KW0007-Acetylation, FDRq = 2.64e-35) and **(B)**, ubiquitinated (UniProt KW0832-Ubl conjugation, FDRq=6.37e-14) proteins. Proteins annotated as Acetylation and Ubl conjugation are indicated in green and purple, respectively. DC proteins that were also quantified in the EVs are outlined in yellow. Proteins are labeled according to gene name.

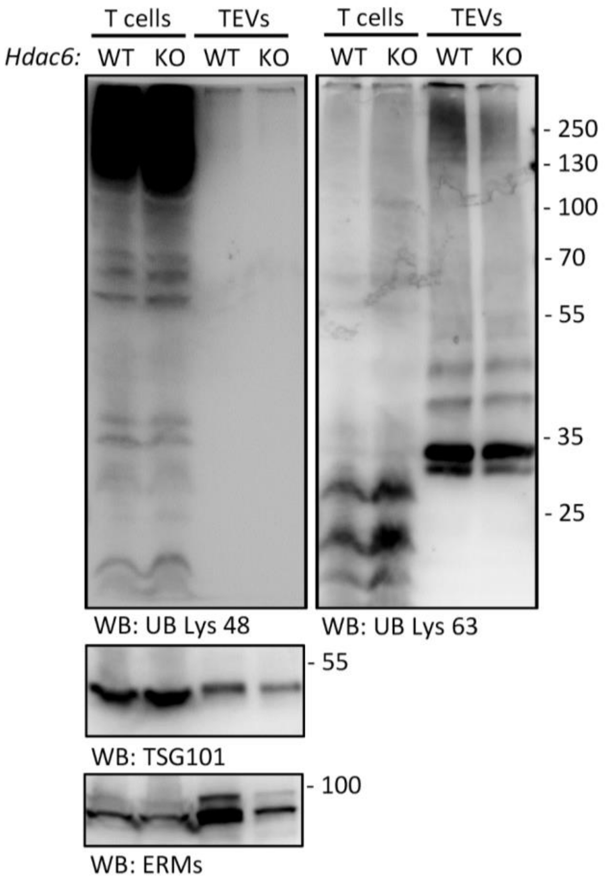

Supplementary Figure 4. Ubiquitination in K-48 and K-63 state in T lymphoblast total cell lysates and their derived EVs. Protein extracts were prepared from EVs and *Hdac6*$^{+/+}$ (WT) or *Hdac6*$^{-/-}$ (KO) T cell lymphoblasts samples isolated from spleen. Cells and EVs were blotted with an antibody against polyubiquitin chains linked through Lys48 ubiquitin (UB) residue (left panel) or Lys63 UB residue (right panel). TSG101 and ERMs were used as EVs markers. Both panels corresponds to same sample.

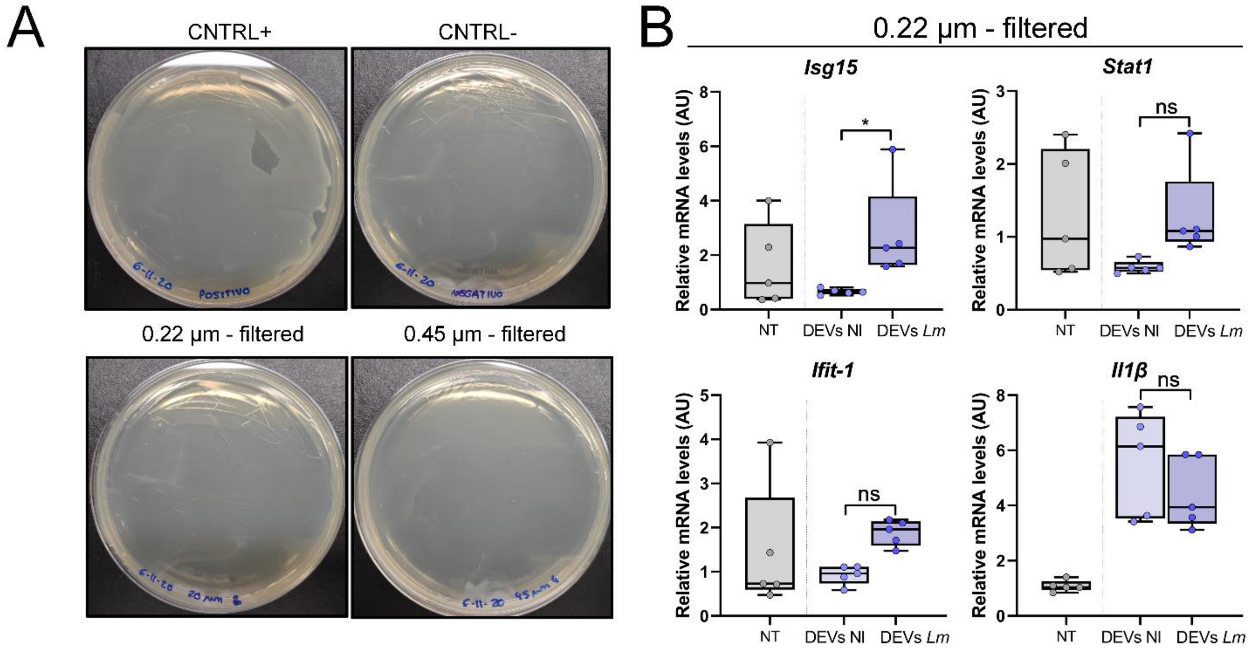

Supplementary Figure 5. Pore filtration methods restrain *Lm* and do not induce strong anti-pathogenic responses. (A) BHI-Agar plates with *Lm* BHI liquid culture (CNTRL+, upper left image), BHI medium (CNTRL-, upper right image) and *Lm* filtered through 0.22 µm (lower left image) or 0.45 µm (lower right image) cultured for over 3 days**. (B)** qPCR analysis of the expression levels of *Isg15*, *Stat1*, *Ifit-1* and *Il1β* in DCs treated with vehicle (NT), DEVs isolated from non-infected DCs (NI) or *Lm*-infected (Lm). DEVs were filtered through 0.22 µm (*n*=5, one-way analysis of variance (ANOVA) test with Tukey's posttest; *P < 0.05 and ns, not significant).

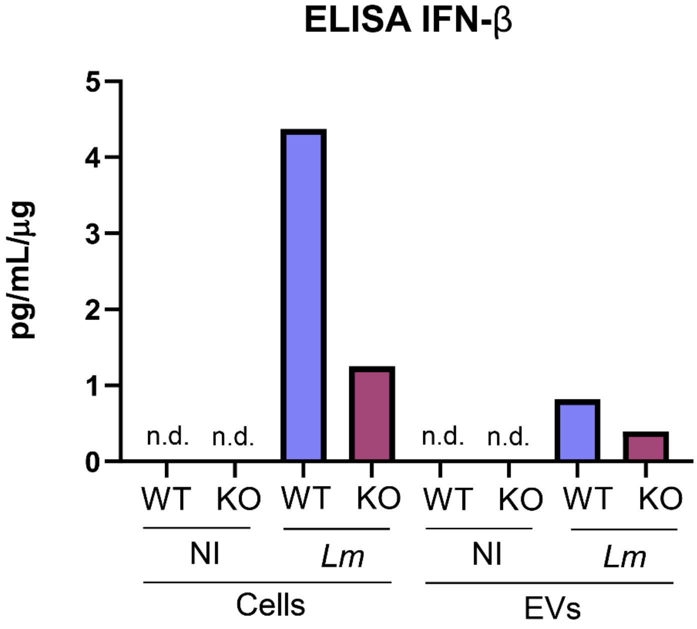

Supplementary Figure 6. IFN-β is detected following *Lm* infection. HDAC6 WT or KO whole DCs (Cells) or dendritic derived extracellular vesicles (EVs) lysate under non-infection (NI), or Lm-infection (*Lm*) conditions were subjected to LumiKine™ Xpress mIFN-β 2.0 assay. Detected pg/mL were plot based on total amount of proteins in the lysate (µg). Non-detectable samples are indicated as n.d.

2.2 Supplementary Tables

**Reagent**	**Application**	**Use**	**Reference**	**Manufacturer**
TOPRO-3	Flow cytometry	1:10000	T3605	Invitrogen
LIVE/DEAD® Fixable Yellow Dead Cell Stain	Flow cytometry	1:1000	L34968	Invitrogen
CD11b-PECy7	Flow cytometry	1:200	25-0112-81	Invitrogen
CD11c-BV421	Flow cytometry	1:200	565452	BD
GR-1-V450	Flow cytometry	1:200	75-5931	Tonbo Biosciences
MHCII-APCFire750	Flow cytometry	1:400	107652	BioLegend
Annexin A2	Western-blot	1:1000	ab185957	Abcam
Annexin A6	Western-blot	1:1000	686921	R&D Systems
Calnexin	Western-blot	1:500	ab10286	Abcam
ERMs	Western-blot	1:4000		Rabbit ascitis
NLRP3/NALP3	Western-blot	1:500	AG-20B-0014-C100	AdipoGen Life Sciences
MFG-E8	Western-blot	1:500	AF2805	R&D Systems
RIG-I	Western-blot	1:500	3743S	Cell Signaling Technology
STAT1	Western-blot	1:1000	9172	Cell Signaling Technology
TSG101	Western-blot	1:1000	ab83	Abcam
UB Lys 48	Western-blot	1:500	ab140601	Abcam
UB Lys 63	Western-blot	1:1000	05-1308	Sigma-Aldrich
α-Tubulin	Western-blot	1:1000	T6199	Sigma-Aldrich
Anti-mouse-HRP	Western-blot	1:5000	31446	Invitrogen
Anti-rabbit-HRP	Western-blot	1:5000	31460	Invitrogen
Anti-goat-HRP	Western-blot	1:5000	31402	Invitrogen

Table S1. List of antibodies used for Western-blot and Flow Cytometry and the used dilution.

Gene	Forward Sequence	Reverse Sequence
Ywhaz	CGTTGTAGGAGCCCGTAGGTCAT	TCTGGTTGCGAAGCATTGGG
β-Actin	CAGAAGGAGATTACTGCTCTGGCT	TACTCCTGCTTGCTGATCCACATC
Isg15	CTAGAGCTAGAGCCTGCAG	AGTTAGTCACGGACACCAG
Stat1	CGCGCATGCAACTGGCATATAACT	ATGCTTCCGTTCCCACGTAGACTT
Ifit1	CTGAGATGTCACTTCACATGGAA	GTGCATCCCCAATGGGTTCT
Il1b	AAAGACGGCACACCCACCCTGC	TGTCCTGACCACTGTTGTTTCCCAG
Il12 p40	TGGTTTGCCATCGTTTTGCTG	ACAGGTGAGGTTCACTGTTTCT
Cd40	GACTCAGGCGAATTCTCAGC	GTCTCAGTGGCCATCTCCAT
Cd86	CTGGACTCTACGACTTCACAATG	AGTTGGCGATCACTGACAGTT
Cxcl10	CCAGTGCTGCCGTCATTTTC	GGCTCGCAGGGATGATTTC

Table S2. List of primers, with their corresponding sequence, used for qPCR.

Table S3: Protein quantification in total cell lysates

Table S4: IPA analysis of total cell lysates: canonical pathways and diseases and functions category

Table S5: Protein quantification in EVs

Table S6: IPA analysis of EVs: diseases and functions category

Table S7: Ubiquitinated and acetylated peptides in total cell lysates and EVs

Table S8: Enrichment analysis of ubiquitinated and acetylated proteins
